# The Role of Crosstalk Between the Unfolded Protein Response and Autophagy in Diseases Associated with Sympathetic Nervous System Imbalance: Mechanisms and Therapeutic Perspectives

**DOI:** 10.3390/ijms27031282

**Published:** 2026-01-27

**Authors:** Bo Xu, Yi Yang, Renjun Wang

**Affiliations:** Department of Biotechnology, School of Life Science, Jilin Normal University, Siping 136000, China; xubo@mails.jlnu.edu.cn

**Keywords:** sympathetic nervous system imbalance, unfolded protein response, autophagy, endoplasmic reticulum stress, metabolic diseases

## Abstract

Sympathetic nervous system (SNS) imbalance is a common pathological basis for cardiovascular diseases, non-alcoholic fatty liver disease, and diabetes. This review focuses on these diseases, analyzing two core mechanisms: excessive sympathetic excitation induced by endoplasmic reticulum stress (ERS) or autophagy dysfunction in key central nuclei (e.g., hypothalamus, rostral ventrolateral medulla); and ERS/autophagy abnormalities in peripheral target organs caused by chronic SNS overactivation. Existing studies confirm that chronic SNS overactivation promotes peripheral metabolic overload via sustained catecholamine release, inducing persistent ERS and disrupting the protective unfolded protein response (UPR)–autophagy network, ultimately leading to cell apoptosis, inflammation, and fibrosis. Notably, central ERS or autophagy dysfunction further perturbs autonomic homeostasis, exacerbating sympathetic overexcitation. This review systematically elaborates on SNS overactivation as a critical bridge mediating UPR–autophagy network dysregulation in central and peripheral tissues, and explores therapeutic prospects of targeting key nodes (e.g., chemical chaperones, specific UPR modulators, nanomedicine), providing a theoretical basis for basic research and clinical translation.

## 1. Introduction

The high prevalence of major diseases such as chronic heart failure, obesity, and non-alcoholic fatty liver disease has become a global public health challenge. Their pathological mechanisms are closely associated with disruptions in cellular homeostasis and dysfunction of the sympathetic nervous system. Cellular homeostasis is fundamental to maintaining normal physiological functions. The endoplasmic reticulum (ER), a central site for protein synthesis and lipid metabolism, can develop functional disturbances leading to endoplasmic reticulum stress (ERS). The unfolded protein response (UPR) is activated through three sensors, including inositol-requiring enzyme 1 (IRE1), protein kinase RNA-like endoplasmic reticulum kinase (PERK), and activating transcription factor 6 (ATF6), and functions to restore ER homeostasis [1,2,3]. Autophagy maintains metabolic balance by degrading damaged organelles and abnormal proteins and is transcriptionally regulated by transcription factor EB (TFEB). Its functional impairment can lead to disorders such as insulin resistance and hepatic steatosis [4,5,6]. Close crosstalk exists between the UPR and autophagy. All three branches of the UPR can activate autophagy. Furthermore, spliced X-box binding protein 1 (sXBP1) can drive autophagy-related transcriptional regulation mediated by TFEB. Together, they form a core network for maintaining cellular metabolic balance [7,8,9].

The sympathetic nervous system (SNS) regulates physiological functions such as heart rate, blood pressure, and metabolism through its multi-system innervation [10,11,12,13]. Under physiological conditions, it maintains a dynamic balance with the parasympathetic nervous system. Persistent stress or pathological stimulation can lead to excessive SNS activation [14,15]. An imbalance in central signaling is a key factor driving major diseases associated with the sympathetic nervous system. This dysregulation is particularly prevalent in critical nuclei such as the hypothalamic paraventricular nucleus (PVN), the subfornical organ (SFO), and the rostral ventrolateral medulla (RVLM). The core mechanism involves a reduction in central inhibitory regulation, which ultimately leads to excessive enhancement of sympathetic nerve activity. This further underscores the central anatomical positioning and critical physiological regulatory significance of these brain regions in the initiation and progression of sympathetic-related diseases [16,17,18]. Sympathetic imbalance also promotes cardiac structural and functional deterioration through mechanisms such as dysregulated β-adrenergic receptor signaling and abnormal cAMP compartmentalization [19]. In metabolic diseases like obesity, non-alcoholic fatty liver disease (NAFLD), and diabetes, excessive SNS activation similarly constitutes a core pathological link in inducing metabolic disorders [20,21].

Although the association between SNS imbalance and multi-organ diseases is well-established, the linking molecular pathways remain unclear. Recent studies indicate that excessive SNS activation contributes to disrupted cellular homeostasis by remodeling UPR–autophagy crosstalk, supported by preclinical evidence that SNS blockers such as stellate ganglion block and β-adrenergic receptor antagonists reverse ER stress and restore autophagic flux. However, direct causal mechanisms remain undefined. A key unanswered question is whether catecholamines bind UPR sensors or autophagy regulators. These mechanisms require validation via cell-specific knockout or conditional activation studies [22,23]. Chronic SNS activation leads to sustained catecholamine release, which induces metabolic overload and persistent ERS in target organ cells. This shifts the UPR–autophagy network toward pro-apoptotic and pro-inflammatory programs—a pathological transition that has been validated in diseases of organs such as the liver and heart [20,24,25,26]. However, a complete theoretical framework is currently lacking with regard to two core mechanisms: (1) how central UPR initiates SNS activation and drives peripheral pathologies, and (2) how SNS imbalance subsequently regulates the disorder of organ-specific UPR–autophagy networks.

This review focuses on the mechanistic role and therapeutic prospects of the UPR–autophagy crosstalk in SNS imbalance-related diseases. We will systematically outline the molecular regulation and intersectional mechanisms of ERS, the UPR, and autophagy. Specifically, this review will dissect the molecular pathways through which excessive SNS activation disrupts the UPR–autophagy network in peripheral target organs and elucidate the central mechanisms (e.g., in the hypothalamic paraventricular nucleus, PVN) where ERS or autophagy dysfunction triggers sympathetic excitation. By integrating evidence from cardiovascular and metabolic diseases, the review will summarize the disease-specific manifestations of this dysregulated network. Furthermore, it will explore the application prospects and translational potential of therapeutic strategies targeting key nodes of the UPR–autophagy axis, such as chemical chaperones and specific UPR modulators. Ultimately, this review aims to synthesize existing evidence to construct a theoretical framework linking SNS imbalance, dysregulated UPR–autophagy crosstalk, and multi-organ pathology, thereby providing a foundation for basic research and the development of novel therapeutic strategies for related diseases. Notably, the temporal sequence between SNS overactivation and ER stress—i.e., whether central ER stress precedes sympathetic excitation across all disease contexts, or if peripheral ER stress can feed back to drive central sympathetic activation—remains incompletely elucidated.

## 2. Synergistic Strategies in Cellular Quality Control: Endoplasmic Reticulum Stress-Mediated Selective Autophagy and Metabolic Remodeling

Cellular homeostasis in protein and organelle function is fundamental to organismal physiology. ERS, triggered by ER dysfunction, initiates the regulatory UPR. Conversely, autophagy maintains homeostasis by clearing damaged components. These two systems form a crosstalk network, working synergistically to preserve stability under physiological conditions. Their dysregulation, however, drives disease progression under pathological conditions.

### 2.1. Core Pathways of the UPR

When unfolded or misfolded proteins accumulate in the endoplasmic reticulum, they activate three stress sensors located on the ER membrane: IRE1, PERK, and ATF6. This activation initiates three distinct yet interconnected signaling pathways. Together, they work to restore ER functional homeostasis by regulating protein synthesis, folding, and degradation.

#### 2.1.1. The IRE1α-XBP1 Pathway: Regulating Folding, Degradation, and Lipid Synthesis

IRE1α is the most evolutionarily conserved signaling branch of the UPR. As a transmembrane protein on the ER membrane, it possesses dual activities of serine/threonine kinase and endoribonuclease [27,28]. Upon ERS, IREα senses unfolded proteins, leading to its oligomerization and autophosphorylation, which activates its endoribonuclease activity. This activity drives two primary downstream processes. First, it specifically splices the mRNA of X-box binding protein 1 (XBP1), generating the transcriptionally active sXBP1 [29,30,31]. sXBP1 translocates to the nucleus and upregulates genes encoding ER chaperones and foldases, thereby enhancing protein folding capacity [32]. Second, it initiates the regulated IRE1-dependent decay (RIDD) pathway to degrade mRNAs encoding secreted proteins, thereby reducing the ER’s folding burden [31]. Notably, the function of IRE1α is context-dependent: beyond its protective effects, sustained ERS can activate its kinase activity to trigger the JNK pro-inflammatory pathway or degrade mRNAs of anti-apoptotic genes (e.g., Bcl-2) via RIDD, promoting cell apoptosis [31]. In NAFLD, hyperactivation of the IRE1α-XBP1 pathway exacerbates lipid accumulation by inducing fatty acid synthase expression via sXBP1, rather than merely maintaining lipid homeostasis [32,33,34,35]. An isoform, IRE1β, exists in the intestine where it regulates mucin production and secretion [36,37]. However, in the context of SNS imbalance-related metabolic and cardiovascular diseases, the IRE1α–XBP1 pathway plays a more central role.

#### 2.1.2. The PERK–eIF2α–ATF4 Pathway: Regulating Translation, Oxidative Stress, and Autophagy

PERK is an ERS sensor. Under stress conditions, it activates via oligomerization and autophosphorylation. Its primary downstream target is eukaryotic initiation factor 2α (eIF2α) [38]. Phosphorylation of eIF2α by PERK inhibits global protein translation initiation, reducing the influx of newly synthesized peptides into the ER and mitigating folding pressure [39,40]. This translational inhibition is selective; conversely, phosphorylated eIF2α promotes the translation of activating transcription factor 4 (ATF4) mRNA. As a key transcription factor, ATF4 regulates downstream target genes. Specifically, it induces the expression of antioxidant enzymes, such as heme oxygenase-1, under oxidative stress to enhance cellular antioxidant capacity [41]. In the context of autophagy regulation, it directly binds to promoters of autophagy-related genes like *Atg5* and *LC3*, promoting their transcription to initiate autophagy [42,43]. Furthermore, ATF4 induces the expression of C/EBP-homologous protein (CHOP). CHOP participates in adaptive responses during mild stress but triggers apoptosis under sustained stress, serving as a critical molecular switch for the physiological-to-pathological functional transition of this pathway [44].

#### 2.1.3. The ATF6 Pathway: Transcriptional Regulatory Functions

ATF6 is a type II transmembrane transcription factor located on the ER membrane. Under resting conditions, it is kept inactive by binding to the ER chaperone protein binding immunoglobulin protein (BiP). Upon the accumulation of unfolded proteins in the ER, BiP dissociates from ATF6, allowing ATF6 to translocate to the Golgi apparatus [45]. Within the Golgi, ATF6 is sequentially cleaved by the proteases S1P and S2P. This process generates an active cytosolic fragment (ATF6f), which enters the nucleus to exert transcriptional regulation [46]. ATF6f primarily targets genes encoding ER chaperones and components of the ER-associated degradation (ERAD) system. By enhancing protein-folding capacity and the efficiency of misfolded protein clearance, it alleviates ER stress [47]. Clinical studies indicate that the ATF6 pathway is activated in the intestinal tissues of patients with inflammatory bowel disease, where it upregulates BiP to mitigate ER stress during chronic inflammation [48]. In metabolic disorders such as non-alcoholic fatty liver disease (NAFLD), abnormal activation of the ATF6 pathway is involved in regulating hepatic lipid metabolism dysregulation [49]. Furthermore, ATF6 cooperates with XBP1s to coordinately regulate ER stress-related genes, forming a collaborative network among different UPR pathways [50]. The protective and detrimental effects of the PERK-eIF2α-ATF4 pathway depend on the intensity of stress: under mild stress, ATF4 initiates protective autophagy by inducing *Atg5* and LC3 [42,43]; whereas under sustained stress, excessive activation of ATF4 upregulates CHOP, ultimately triggering cell apoptosis [44]. In myocardial ischemia/reperfusion injury, hyperactivation of this pathway also drives excessive autophagy, leading to cardiomyocyte loss [51].

### 2.2. Core Process, Types, and Regulation of Autophagy

Autophagy is a conserved process by which cells degrade damaged organelles, misfolded proteins, and metabolic waste via lysosomes, maintaining intracellular homeostasis and contributing to cell survival and stress adaptation. Its core process includes initiation, nucleation, elongation, fusion, and degradation [52].

Autophagic flux describes the complete autophagic process—encompassing autophagosome formation, fusion with lysosomes, and substrate degradation—and reflects autophagic functional activity. Autophagosome accumulation is a phenotypic observation of increased autophagosome numbers. It can arise from two scenarios: enhanced autophagic flux with increased formation and normal degradation, or blocked autophagic flux with impaired fusion or degradation leading to buildup. Distinguishing between the two relies on combined detection of autophagy markers [53]; enhanced autophagic flux is defined by increased expression of the autophagosome marker LC3-II and decreased expression of p62/SQSTM1—a substrate marker degraded through autophagy. Blocked autophagic flux is characterized by increased LC3-II expression alongside elevated p62/SQSTM1, resulting from substrate accumulation due to impaired degradation.

#### 2.2.1. Core Process and Regulation of Macroautophagy

Macroautophagy is the most characteristic form of autophagy, primarily responsible for degrading large organelles such as mitochondria and the ER. Its orderly progression relies on precise coordination at multiple key regulatory nodes. The phosphorylation status of the unc-51-like autophagy activating kinase 1 (ULK1) complex directly determines autophagy initiation. Under nutrient starvation or stress conditions, AMPK directly phosphorylates ULK1 at Ser317/777 sites, thereby relieving inhibition by mTOR and initiating the autophagy cascade [54]. The Beclin1–VPS34 complex catalyzes the generation of PI3P to recruit downstream ATG proteins. Concurrently, ATG9-mediated vesicle trafficking supplies the necessary membrane sources for phagophore formation, promoting autophagosome biogenesis [55,56]. The LC3 lipidation system, involving ATG7 and ATG3, facilitates the conjugation of LC3 to the autophagosome membrane, making it a core marker for autophagosomes and a key molecule for substrate recognition [57]. Recent studies have further revealed that the transcription factor TFEB upregulates the expression of ATG genes such as *LC3* and *Beclin1*. This effectively enhances autophagic flux during lysosomal dysfunction, establishing TFEB as a crucial target in the field of transcriptional regulation of autophagy [58].

#### 2.2.2. Other Major Types of Autophagy: Chaperone-Mediated Autophagy and Microautophagy

Chaperone-mediated autophagy (CMA) specifically degrades soluble proteins. It involves the recognition of substrates by HSPA8 (Hsc70), their binding to the lysosomal membrane protein lysosome-associated membrane protein type 2A (LAMP2A), and subsequent translocation across the lysosomal membrane for degradation [59]. CMA plays a unique role in protein quality control, and its functional decline is closely associated with aging and neurodegenerative diseases [60]. Microautophagy involves the direct engulfment of cytoplasmic components by invagination of the lysosomal membrane. It can be categorized into selective degradation via microautophagic bodies and non-selective microautophagy. This process primarily participates in lipid droplet degradation and organelle turnover, regulating lipid accumulation in hepatic lipid metabolism [61,62,63]. Unlike macroautophagy, both CMA and microautophagy do not require the formation of a double-membrane autophagosome, instead relying on the remodeling of the lysosomal membrane itself. Together, these three pathways constitute the cellular degradation network [64].

#### 2.2.3. Physiological and Pathological Significance of Autophagy

Under physiological conditions, basal autophagy participates in organelle turnover, metabolic regulation, and immune defense. During embryonic development, autophagy promotes tissue remodeling by degrading excess cellular structures. Genetic knockout of *Atg5* is embryonically lethal in mice [65]. Under pathological conditions, dysfunctional autophagy is closely linked to various diseases. Insufficient autophagy leads to protein aggregation and organelle accumulation, promoting neurodegenerative diseases and tumorigenesis [66,67]. Conversely, excessive autophagy can trigger autophagic cell death, contributing to myocardial ischemia/reperfusion injury and hepatotoxicity [68]. Furthermore, autophagy plays a dual role in metabolic diseases. Specifically, moderate autophagy relieves insulin resistance, whereas chronically high-fat diet-induced excessive autophagy exacerbates lipotoxicity in hepatocytes [69,70]. Notably, autophagy plays a biphasic role in chronic SNS activation-related diseases. Moderate autophagy exerts protective effects by clearing damaged components, while excessive or maladaptive autophagy fails to restore homeostasis and instead triggers pathological cell loss [71,72]. Excessive autophagy refers to abnormally enhanced autophagic flux exceeding cellular adaptive capacity, leading to excessive degradation of essential proteins or organelles and inducing autophagic cell death. Maladaptive autophagy denotes impaired autophagic quality such as incomplete substrate degradation or abnormal autophagosome–lysosome fusion under chronic stress, amplifying cellular damage and promoting apoptosis or necrosis.

### 2.3. Crosstalk Between UPR and Autophagy: Molecular Bridges

The crosstalk regulatory network between the UPR and autophagy exhibits organ- and disease-specific patterns, influencing pathogenesis through the activation or inhibition of pathways such as PERK–ATF4 and IRE1–JNK. When different organs face pathological stimuli, these regulatory axes can exert protective effects. However, under persistent stress, they may switch to destructive mechanisms, thereby driving disease progression (Table 1). It is important to emphasize that the dual nature of individual UPR branches (PERK and IRE1α) plays a critical role in disease progression. While moderate activation of these branches contributes to UPR–autophagy crosstalk and cellular homeostasis, their prolonged signaling can independently drive apoptosis and inflammation, decoupling from protective autophagic responses. This independent pathological role is distinct from the collaborative UPR–autophagy dysregulation and is widely observed in cardiovascular and metabolic diseases [9,28,44].

#### 2.3.1. Diabetic Nephropathy: The ATF4–HO-1 Axis Regulates Autophagy to Maintain Podocyte Homeostasis

In diabetic nephropathy, the UPR pathway plays a central role in podocyte protection by regulating autophagy. A high-glucose environment activates ERS, significantly upregulating ATF4 expression. In turn, ATF4 transcriptionally induces the expression of heme oxygenase-1 (HO-1). HO-1, initiated by ATF4, enhances autophagic flux, thereby clearing damaged organelles and inhibiting podocyte apoptosis—a process defined as protective autophagy [73]. Silencing of the ATF4 gene not only aggravates apoptosis but also blocks the HO-1-mediated protective autophagic response, confirming that the ATF4–HO-1–autophagy axis is a critical pathway for maintaining podocyte homeostasis.

Animal studies demonstrate that the HO-1 agonist hemin can significantly increase renal autophagy levels, reduce proteinuria, and ameliorate glomerulosclerosis. This mechanism provides a novel therapeutic strategy targeting ATF4/HO-1 for diabetic nephropathy.

#### 2.3.2. Liver Disease: Core Mechanisms of UPR-Regulated Autophagy Dysfunction in Metabolic and Toxic Injury

An imbalanced UPR–autophagy network is a common mechanism in the development of liver diseases. In chronic MASLD, suppressed ATF4/CEBPG expression impairs RETREG1-mediated reticulophagy—an adaptive process that normally initiates selective ER-phagy to maintain homeostasis [74]. This defect blocks autophagic flux, causes autophagosome accumulation due to impaired degradation, and fails to attenuate persistent ERS, ultimately exacerbating lipid accumulation and inflammation. This failure to attenuate persistent ERS exacerbates lipid accumulation and inflammation.

In drug-induced liver injury, triptolide excessively activates the PERK–ATF4–CHOP pathway, driving pathological overactivation of autophagy. Conversely, catalpol inhibits this pathway to correct the autophagic imbalance [75]. Alcoholic and non-alcoholic liver diseases share a core link of ERS and autophagy dysfunction. Specifically, persistent ERS disrupts autophagic function, and impaired autophagy, in turn, aggravates ERS. This forms a vicious cycle that promotes fibrosis progression [77]. Targeting liver-specific nodes in the UPR–autophagy crosstalk has emerged as a novel therapeutic strategy for liver diseases.

#### 2.3.3. Heart Disease: Dual Role of the UPR–Autophagy Network in Ischemic Repair and Chronic Failure

The interaction between the UPR and autophagy in heart disease exhibits dynamic evolution. During the acute ischemic phase, vascular endothelial growth factor A (VEGF-A) promotes angiogenesis via the reactive oxygen species (ROS)–ERS–autophagy axis, constituting an adaptive repair mechanism [71]. In contrast, during chronic or excessive stress, the network shifts toward pathological activation. Thrombospondin-1 (Thbs1) aberrantly activates the PERK–ATF4 pathway, driving excessive autophagy and leading to myocardial atrophy [51]. Following myocardial infarction, adenosine triphosphate (ATP) exacerbates reperfusion injury through the P2X7–NADPH oxidase 4 (Nox4)–PERK–ATF4 cascade [76].

In chronic heart failure, autoantibodies against the β1-adrenergic receptor (β1-AR) persistently activate ERS while simultaneously inhibiting autophagic flux. This “activation/inhibition” decoupling disrupts cellular homeostasis [72]. In myocardial ischemia/reperfusion injury, ERS-induced autophagy is initially protective, with CHOP serving as a critical node regulating the balance between autophagy and apoptosis [54]. These findings reveal a dynamic transition of the UPR–autophagy network from a compensatory to a decompensated state in cardiac pathologies.

#### 2.3.4. Pancreatic Islets: Defensive Mechanisms of UPR and Autophagy and the Regulatory Role of CD47

Pancreatic β-cell homeostasis is critical for insulin secretion, and its dysfunction is closely linked to diabetes. Physiologically, UPR alleviates endoplasmic reticulum stress, while autophagy clears abnormal components, together forming a defensive barrier for β-cells. In diabetes, their dysfunction causes β-cell injury [78].

CD47 and its ligand TSP1 are upregulated under metabolic stress, which is also confirmed in clinical samples to be associated with diabetes. Inhibiting CD47 signaling improves UPR function, restores autophagic homeostasis, reduces β-cell senescence and apoptosis, and maintains insulin secretion. The CD47-TSP1 axis is key in regulating islet stress injury, and targeted intervention provides a new direction for diabetes treatment [79].

## 3. Central ERS Initiates SNS Excitation and Drives Peripheral Pathologies

An imbalance in the ERS and autophagy network within key nuclei of the central nervous system acts as a primary driver of SNS excitation. This dysregulation modulates neuronal circuit excitability, leading to excessive activation of the sympathetic centers and subsequently mediating the development of peripheral pathologies. Current research on the association between autophagic/ERS defects in central nuclei and sympathetic excitability primarily involves targeted intervention on key molecules in autophagy/ERS and detecting changes in peripheral sympathetic nerve activity markers, thereby confirming a causal link between the two [80,81,82]. However, these studies have not yet clarified how autophagic/ERS defects directly regulate the excitability of sympathetic neurons. The related molecular mechanisms still require further validation through techniques such as electrophysiological recordings and neuron-specific conditional knockout.

### 3.1. Neurogenic Hypertension: ERS and Autophagy Dysfunction in the RVLM as the Primary Central Driver of Sympathetic Overexcitation and Elevated Blood Pressure

**Figure 1:**
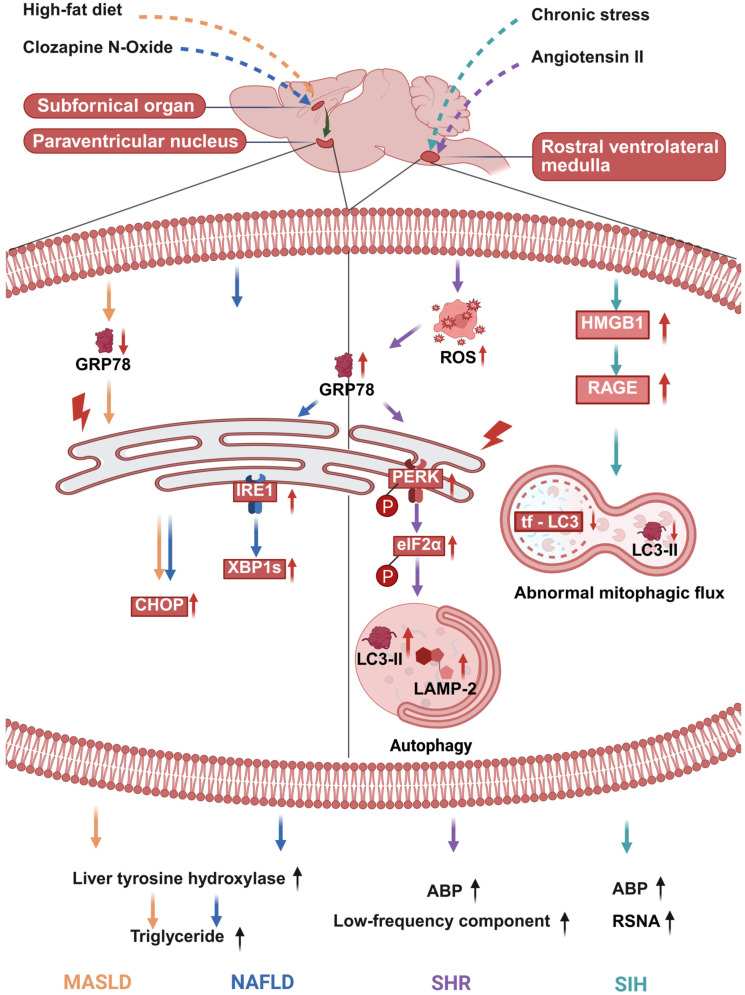
Brain ERS/autophagy initiates sympathetic nervous system (SNS) hyperactivity and drives peripheral lesions. Glucose-regulated protein 78 kDa (GRP78) expression reflects endoplasmic reticulum stress (ERS). GRP78 upregulation signals that cells are counteracting ERS, indicating activated ERS but preserved cellular regulatory capacity. Adequate upregulation reduces unfolded proteins to attenuates ERS, whereas insufficient upregulation drives the progression to decompensation. In contrast, GRP78 downregulation signals the loss of cellular ability to combat ERS, which is accompanied by persistent and aggravated ERS, endoplasmic reticulum (ER) dysfunction, upregulation of pro-apoptotic molecules such as C/EBP homologous protein (CHOP), and ultimately leads to cell injury or death. Low-frequency component, it is a classic experimental indicator reflecting sympathetic neurogenic vasomotor activity. Black dotted lines within the cell membrane are used to separate the two functional parts of the figure: the left part represents PVN-associated processes, while the right part represents RVLM-associated processes. In this figure, “↑” indicates an increase in expression or activity of the corresponding molecule/pathway; meanwhile, arrows of different colors represent distinct regulatory pathways (e.g., the orange dashed arrow corresponds to the high-fat diet pathway, etc.). Created in BioRender. Bo Xu, Renjun Wang. (2025) https://BioRender.com/w55g70q (accessed on 18 December 2025). Abbreviations: ABP (arterial blood pressure); CHOP (C/EBP homologous protein); eIF2α (eukaryotic translation initiation factor 2 subunit alpha); GRP78 (glucose-regulated protein 78); HMG B1 (high-mobility group box 1); IRE1 (inositol-requiring enzyme 1); LC3-II (microtubule-associated protein 1A/1B-light chain 3-II); LAMP-2 (lysosomal-associated membrane protein 2); MASLD (metabolic dysfunction-associated steatotic liver disease); NAFLD (non-alcoholic fatty liver disease); PERK (protein kinase RNA-like endoplasmic reticulum kinase); RAGE (receptor for advanced glycation end products); ROS (reactive oxygen species); RSNA (renal sympathetic nerve activity); SHR (spontaneously hypertensive rat); SIH (stress-induced hypertension); XBP1s (spliced X-box binding protein 1).

The RVLM is the core central site for regulating sympathetic cardiovascular tone. ERS and autophagy dysfunction within the RVLM serve as a primary central mechanism for neurogenic hypertension. In the spontaneously hypertensive rat (SHR) model, markers of ERS in the RVLM are significantly upregulated even before the onset of the hypertensive phenotype. Notably, a bidirectional regulatory relationship exists between oxidative stress and ERS. Specifically, angiotensin II (Ang II)-induced oxidative stress can trigger ERS in the RVLM, whereas scavenging ROS or stabilizing ER homeostasis can reverse the hypertensive phenotype. Concurrently, autophagy in the RVLM of SHRs is overactivated, as indicated by increased expression of lysosome-associated membrane protein 2 (LAMP-2) and LC3-II. Importantly, this excessive autophagy is dependent on ERS initiation. Inhibiting autophagy or silencing the *LC3-II* gene in the RVLM can produce a blood pressure-lowering effect. In summary, the oxidative stress-sensitive ERS and the consequent autophagy dysregulation in the RVLM jointly drive sympathetic overexcitation and elevated blood pressure, constituting the core central mechanism of neurogenic hypertension (Figure 1) [80].

### 3.2. Heart Failure: Central Nuclei-Mediated Sympathetic Imbalance via ERS and Signaling Pathways

In volume overload-induced high-output heart failure, RVLM catecholaminergic neuron activation causes autonomic imbalance. RVLM exhibits significant ERS, inflammation, and renin-angiotensin system activation, elevating the LFHRV/HFHRV ratio (sympathetic overactivation marker). Intracerebroventricular administration of ERS inhibitor TUDCA abolishes RVLM ERS, reduces arrhythmias and apneas, and alleviates cardiac hypertrophy and diastolic dysfunction, confirming RVLM ERS as a key driver of sympathetic imbalance-mediated cardiorespiratory dysfunction [83].

In myocardial infarction-induced HF, hypothalamic PVN epidermal growth factor receptor (EGFR) phosphorylation increases, activating the ERK1/2 pathway. This axis promotes PVN neuroinflammation (as reflected by elevated TNF-α and IL-1β levels), enhances RAS activity, and induces ERS (marked by increased BIP and ATF4 expression), enhancing sympathetic outflow and elevating norepinephrine levels. PVN microinjection of EGFR siRNA downregulates p-EGFR/p-ERK1/2, suppresses pathological pathways, and improves HF phenotypes, highlighting PVN EGFR-ERK1/2 signaling in sympathetic excitation [84].

### 3.3. Obesity: Hypothalamic POMC Neuron MANF Regulates Sympathetic Activity and BAT Thermogenesis via ERS

Obesity is tightly linked to sympathetic imbalance from hypothalamic dysfunction, with POMC neurons critical for energy homeostasis. Mesencephalic astrocyte-derived neurotrophic factor (MANF) in POMC neurons is a key regulator. MANF deletion in POMC neurons increases diet-induced obesity susceptibility by inducing hypothalamic ERS and leptin resistance, reducing POMC expression/processing, and decreasing sympathetic activity and BAT thermogenesis [85]. Conversely, MANF overexpression alleviates ERS, enhances POMC function, stimulates BAT sympathetic innervation/thermogenesis, and protects against obesity, highlighting MANF’s essential role via the ERS-POMC-sympathetic-BAT axis.

### 3.4. Stress-Induced Hypertension: Targeting the HMGB1/RAGE Axis in the RVLM Restores Mitophagic Flux and Inhibits Sympathetic Overexcitation

Chronic stress can induce the translocation of high-mobility group box 1 (HMGB1) from the nucleus to the cytoplasm and upregulate the expression of its receptor—namely, receptor for advanced glycation end products (RAGE)—in microglia within the RVLM. The activated HMGB1/RAGE axis serves as a key central driver of sympathetic excitation in stress-induced hypertension (SIH). This axis exerts a dual regulatory effect on mitophagic flux in microglia. It initiates autophagy during the early stress phase, but later impairs mitophagic flux by downregulating the expression of RAB7 and LAMP1 and increasing lysosomal pH, thereby compromising lysosomal function. This mitophagic flux blockade sequentially leads to mitochondrial damage, oxidative stress, and M1 polarization of microglia (Figure 1) [86]. Specific knockdown of *HMGB1* or knockout of *RAGE* in the RVLM ameliorates mitophagic flux impairment, inhibits microglial neuroinflammation, and consequently reduces blood pressure and renal sympathetic nerve activity in mice. These findings indicate that targeting the HMGB1/RAGE axis in the RVLM can effectively suppress sympathetic overexcitation and stress-induced hypertension.

### 3.5. Non-Alcoholic Fatty Liver Disease: ERS in the SFO–PVN Circuit Directly Commands Hepatic Metabolic Reprogramming via the Hepatic Sympathetic Nerve

The onset and progression of non-alcoholic fatty liver disease (NAFLD) are closely associated with the regulation of forebrain–hypothalamic neural circuits. Among these, dysfunction of the subfornical organ–paraventricular nucleus (SFO–PVN) circuit serves as a key central mechanism mediating hepatic steatosis. In a normal mouse model, acute chemogenetic activation of SFO–PVN neurons can induce hepatic steatosis in a liver sympathetic nerve-dependent manner. Conversely, inhibiting this forebrain–hypothalamic circuit effectively improves obesity-associated NAFLD pathological phenotypes [81].

Further studies confirm that diet-induced NAFLD is accompanied by ultrastructural alterations in the endoplasmic reticulum (ER) and activation of ERS within the PVN. Notably, these changes can be mitigated by reducing excitatory signaling from the SFO. Similarly, specific inhibition of ERS in PVN neurons also ameliorates hepatic steatosis under obese conditions. In summary, ERS activation within the SFO–PVN circuit can directly regulate hepatocyte metabolic processes via the hepatic sympathetic nerve. This drives hepatic metabolic reprogramming and promotes abnormal lipid accumulation (Figure 1) [81]. The aberrant activation of this circuit constitutes a significant central driver of NAFLD pathogenesis. Targeted intervention against ERS in the SFO–PVN circuit may therefore provide a novel therapeutic direction for NAFLD.

### 3.6. Metabolic Dysfunction-Associated Steatotic Liver Disease: Neuronal ERS in the SFO-PVN Circuit Drives Hepatic Lipid Uptake via the Sympathetic Nerve

The pathological progression of MASLD is closely linked to ERS within the central SFO–PVN neural circuit. In a high-fat diet-induced obese mouse model, ERS in SFO–PVN neurons regulates hepatic lipid metabolism via the sympathetic nervous system. Blocking ERS in the neurons of this circuit not only significantly reduces hepatic triglyceride accumulation and the expression levels of genes related to lipid uptake but also decreases the content of tyrosine hydroxylase—the rate-limiting enzyme for catecholamine synthesis—in the liver. Moreover, the hepatic expression level of tyrosine hydroxylase shows a positive correlation with the lipid uptake pathway, but no significant association with the lipid breakdown pathway [82].

These results confirm that neuronal ERS in the SFO–PVN circuit upregulates hepatic tyrosine hydroxylase expression via the sympathetic nerve. This, in turn, enhances the lipid uptake capacity of hepatocytes, ultimately promoting the development of MASLD. Targeted inhibition of ERS in SFO–PVN neurons can alleviate obesity-associated hepatic steatosis by blocking the sympathetic nerve-driven stimulation of hepatic lipid uptake (Figure 1).

The reviewed evidence establishes central ERS as a convergent driver of sympathetic overexcitation across multiple diseases. A key unanswered question is whether this represents a primary cause or a secondary consequence. Current data best support a “vicious cycle” model that transcends this dichotomy: peripheral insults (hemodynamic, metabolic, or inflammatory) act as initial triggers, activating central ERS via humoral or neural signals. Once engaged, central ERS orchestrates self-amplifying responses—including neuroinflammation and neuronal excitability changes—that lock sympathetic centers into sustained overactivity. This, in turn, worsens peripheral pathology, reinforcing the feedback loop. Thus, in established disease, central ERS evolves from a triggered response into an independent, perpetuating pathological driver. This explains the efficacy of centrally targeted ERS interventions. Furthermore, future research employing temporally controlled, cell-specific manipulations is needed to dissect the precise causal weight of central ERS within this cycle, which is essential for developing stage-specific therapies. In addition, future research should also focus on neuronal subtype specificity in autonomic centers (e.g., glutamatergic and GABAergic neurons in the PVN and RVLM), exploring whether UPR/autophagy pathways exert distinct regulatory effects on different neuronal subtypes to mediate sympathetic overexcitation, which will help further clarify the precise central regulatory network.

## 4. SNS Imbalance Drives Organ-Specific UPR/Autophagy Network Disruption

The disruption of UPR–autophagy networks in peripheral organs by SNS imbalance involves two distinct pathways: direct regulation via adrenergic receptor binding and indirect modulation through secondary metabolic disorders. Below are organ-specific manifestations of these two pathways (Table 2).

### 4.1. Doxorubicin-Induced Heart Failure: Nox2-Mediated Sympathetic Nerve Terminal Dysfunction and Cardiomyocyte Autophagy Lead to Cardiac Atrophy

In wild-type mice injected with doxorubicin, sympathetic nerve terminal damage is prominent. Specifically, myocardial noradrenergic nerve fibers are reduced, and the expression of sympathetic markers such as protein gene product 9.5 (PGP9.5) and growth-associated protein 43 (GAP43) is downregulated. Concurrently, cardiomyocyte autophagy is hyperactivated, leading to reduced cell size and ultimately resulting in cardiac atrophy and functional decline. In contrast, these pathological alterations and doxorubicin-induced cardiac injury are significantly ameliorated in Nox2 knockout mice [87]. Beyond targeted modulation of Nox2, spironolactone (SP) has also been shown to ameliorate doxorubicin-induced myocardial fibrosis, apoptosis, and cardiac dysfunction, representing another potential strategy for protecting against doxorubicin cardiotoxicity [93].

**Figure 2:**
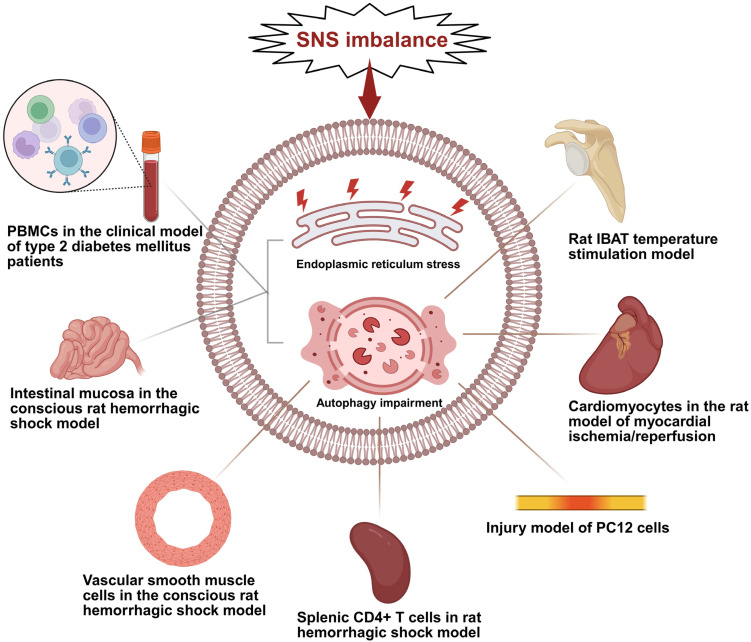
Schematic diagram illustrating SNS imbalance driving organ-specific ERS and autophagy impairment. This diagram summarizes the differential pathological effects of SNS imbalance (the core upstream driver) on target cells/tissues across various models, where gray lines represent the occurrence of both ERS and autophagy impairment in the corresponding cells/tissues, and brown lines represent the occurrence of only autophagy impairment; PBMCs (Peripheral Blood Mononuclear Cells); IBAT (Interscapular Brown Adipose Tissue); PC12 (a rat pheochromocytoma cell line commonly used as a neuronal cell model in in vitro studies). Created in BioRender. Bo Xu, Renjun Wang. (2025) https://BioRender.com/mvdqtdb (accessed on 18 December 2025).

In summary, Nox2 mediates oxidative stress, which triggers sympathetic nerve terminal dysfunction and directly activates cardiomyocyte autophagy (Figure 2). These two processes act synergistically to cause cardiac atrophy and progression to heart failure. Targeting Nox2 to rebalance the sympathetic nerve–autophagy axis provides a central direction for preventing and treating this form of drug-induced cardiotoxicity.

### 4.2. Brown Adipose Tissue Atrophy: The Sympathetic Nerve Maintains Tissue Mass by Inhibiting FoxO-Mediated Autophagy

The SNS serves as a central regulator for maintaining brown adipose tissue (BAT) mass by inhibiting forkhead box O (FoxO)-mediated autophagy. Under physiological conditions, sympathetic activation suppresses autophagy and promotes anabolism to maintain tissue homeostasis. In contrast, a lack of sympathetic tone triggers FoxO-mediated hyperactivation of autophagy, ultimately leading to tissue atrophy.

Experimental evidence shows that sympathetic denervation of the interscapular BAT in rats at 25 °C activates FoxO dephosphorylation. This drives accelerated autophagic flux and an increase in autophagosomes, culminating in tissue atrophy. Conversely, during cold stimulation at 4 °C, the sympathetic nerve is activated. This activation stimulates mechanistic target of rapamycin (mTOR) signaling via the cAMP/Epac1/Akt pathway, which promotes protein synthesis and simultaneously inhibits Unc-51 like autophagy activating kinase 1 (ULK1), a key protein for autophagy initiation, thereby blocking autophagosome formation and ultimately mediating tissue hypertrophy [88]. This mechanism clarifies the pivotal role of the sympathetic nerve–autophagy axis in BAT cold adaptation (Figure 2).

### 4.3. Methamphetamine-Induced Neuronal Injury Model: Norepinephrine Exerts a Protective Effect by Restoring Autophagic Flux via Activation of β2-ARs

Recent studies have confirmed that norepinephrine (NE) directly protects against methamphetamine-induced neuronal injury, an effect independent of in vivo neural circuits. In a PC12 cell model, NE reverses the autophagic dysfunction caused by methamphetamine through specific activation of plasma membrane β2-adrenergic receptors (β2-ARs) [22].

Mechanistically, methamphetamine disrupts the autophagic process, causing a blockage in autophagic flux and abnormal diffusion of LC3 protein from autophagic vesicles into the cytoplasm. Agonism of β2-ARs by NE restores autophagic flux and maintains the normal localization of LC3 to autophagic vesicles, thereby repairing autophagic function and reducing cellular damage [22]. This finding establishes a direct neuroprotective pathway: NE signaling → β2-ARs → autophagy regulation. It provides a new perspective for understanding the mechanisms of autophagy dysfunction in NE deficiency-associated neurodegenerative diseases (Figure 2). Notably, other adrenergic receptor subtypes mediate sympathetic regulation of ERS/autophagy: β1-AA (β1-AR ligand) induces myocardial injury, reversed by RD808 via enhancing protective autophagy; β3-AR activation reduces adipose GRP78 (ERS marker) and alleviates metabolic disorders. This reflects a direct adrenergic signaling effect, as NE directly binds to β2-AR on PC12 cells to restore autophagic flux, independent of systemic metabolic changes [22,94,95,96].

### 4.4. Organ Dysfunction Following Hemorrhagic Shock: Stellate Ganglion Blockade Achieves Multi-Target Protection by Inhibiting Autophagy/ERS

During hemorrhagic shock, the SNS is intensely activated. The massive release of catecholamines induces ERS and abnormal autophagy in multiple organs, leading to suppressed immune function, decreased vascular reactivity, and intestinal barrier injury.

#### 4.4.1. Suppressed Splenic Immune Function: SGB Restores CD4^+^ T Cell Function by Inhibiting PHSML-Mediated Autophagy

Hemorrhagic shock induces the production of post-hemorrhagic shock mesenteric lymph (PHSML) via sympathetic activation. PHSML, in turn, activates autophagy in CD4^+^ T cells by inhibiting the PI3K/Akt pathway, resulting in impaired cell proliferation and immune function. Stellate ganglion blockade (SGB) inhibits sympathetic output, reduces PHSML generation, and blocks this autophagic signaling pathway, thereby restoring the proliferative capacity and cytokine secretion function of CD4^+^ T cells. This mechanism is supported by the following evidence. SGB or the autophagy inhibitor 3-MA can reverse T cell functional suppression, whereas the autophagy agonist rapamycin (RAPA) or exogenous PHSML abolishes the protective effect of SGB (Figure 2). Therefore, SGB improves post-hemorrhagic shock immune function by inhibiting the sympathetic nerve–PHSML–autophagy signaling cascade, providing a novel strategy for immunomodulatory therapy [89].

#### 4.4.2. Vascular Hyporeactivity: SGB Maintains Contractile Phenotype by Inhibiting Autophagy-Mediated Phenotypic Switching in Vascular Smooth Muscle Cells

Hemorrhagic shock induces systemic overactivation of the SNS, which subsequently promotes the generation and reflux of PHSML. Acting as a key mediator in sympathetic-tissue crosstalk, PHSML induces excessive autophagy in vascular smooth muscle cells (VSMCs), manifesting as upregulated expression of the autophagic marker proteins LC3-II/I and Beclin1 and aberrant activation of autophagic flux [23].

This autophagic process directly drives phenotypic reprogramming of VSMCs. It switches them from a contractile phenotype, which maintains vascular tone, to a synthetic phenotype characterized by secreting extracellular matrix components and expressing matrix metalloproteinase-2 (MMP-2) [23]. This switch ultimately leads to diminished vascular contractile function and hyporeactivity. By regionally inhibiting sympathetic output, SGB can effectively suppress this process and improve vascular function (Figure 2).

#### 4.4.3. Intestinal Mucosal Barrier Injury: SGB Repairs Barrier Function by Inhibiting Autophagy/ERS

Ischemia, hypoxia, and hypoperfusion induced by hemorrhagic shock lead to blocked autophagic flux (characterized by increased LC3-II and Beclin1, combined with elevated p62 due to impaired degradation) and hyperactivation of the ERS pathway in intestinal mucosa, both of which collectively mediate intestinal mucosal barrier damage. Relevant studies show that SGB, as a sympathetic inhibition strategy, exerts a protective effect by suppressing the ERS pathway in intestinal mucosal cells. In a conscious rat model of hemorrhagic shock, SGB downregulated the abnormal expression of key ERS molecules—ATF6α, PERK, and IRE1α—in intestinal tissue. It also reduced plasma levels of D-lactate and intestinal fatty acid-binding protein. The protective effect of SGB was comparable to that of the ERS inhibitor 4-phenylbutyric acid (4-PBA), whereas the ERS agonist tunicamycin reversed its benefits [90].

Other studies have confirmed that SGB can repair the intestinal mucosal barrier by inhibiting excessive autophagy. Specifically, SGB reversed the hemorrhagic shock-induced upregulation of autophagic markers LC3-II and Beclin1, as well as the downregulation of p62—indicating restored autophagic flux (Figure 2). It also upregulated the expression of tight junction proteins and reduced the intestinal wet-to-dry ratio and plasma fluorescein isothiocyanate–dextran 4 (FD4) levels. The effects of SGB were consistent with those of the autophagy inhibitor 3-methyladenine (3-MA), whereas the autophagy agonist rapamycin (RAPA) counteracted the beneficial effects of SGB. In summary, SGB repairs post-hemorrhagic shock intestinal mucosal barrier function by suppressing sympathetic-driven hyperactivation of both ERS and autophagy [97].

### 4.5. Cardiovascular Risk in Type 2 Diabetes: Sympathetic Tone Drives UPR and Autophagy Pathway Activation in Monocytes

Sympathetic overactivation in type 2 diabetes exerts secondary metabolic stress effects: sustained catecholamine release induces insulin resistance and systemic inflammation, which in turn activate UPR and autophagy pathways in monocytes (Figure 2). This is evidenced by upregulated expression of stress markers such as glucose-regulated protein 78/binding immunoglobulin protein (GRP78/BiP) and phosphorylated EIF2α, as well as activation of the autophagy-related gene BECN1. These changes subsequently trigger inflammatory pathways and oxidative stress responses, promoting the development of atherosclerosis. Clinical studies show that after 4 months of morning-timed treatment with bromocriptine-QR (3.2 mg/day), patients exhibited a significant reduction in sympathetic activity markers. Concurrently, the expression of key ERS/UPR molecules and autophagy genes in monocytes was downregulated, levels of oxidative stress and pro-inflammatory factors decreased, and systemic low-grade inflammation was ameliorated [91]. By inhibiting sympathetic activity and blocking the abnormal activation of this pathway, bromocriptine-QR represents an effective strategy for reducing cardiovascular events.

### 4.6. Arrhythmia: Spinal Nerve Block Restores Cardiac Homeostasis by Inhibiting Sympathetic Overshoot

In a rat model of myocardial ischemia/reperfusion (IR), intrathecal injection of bupivacaine to perform a spinal nerve block inhibited sympathetic overshoot by suppressing abnormal spinal neuronal activity. Spinal nerve block directly inhibits sympathetic overshoot and modulates cardiomyocyte autophagic balance, representing a direct regulation of adrenergic signaling on the cardiac UPR–autophagy network. Simultaneously, it significantly modulated the cardiomyocyte autophagic process and improved autophagic imbalance. This intervention not only reduced the ventricular arrhythmia score and improved heart rate variability but also alleviated IR-induced cardiac dysfunction and myocardial injury. These beneficial effects are closely associated with the restoration of connexin 43 distribution and stabilization of myocardial electrical conduction [92]. Chronic sympathetic overactivation induces sustained β-AR stimulation, driving myocardial UPR dysfunction via the IL-6/gp130/STAT3 pathway. Activated STAT3 (Y705-phosphorylated) specifically triggers PERK and IRE1α, promoting cardiomyocyte apoptosis. Inhibiting this axis reverses cardiac injury in vitro/vivo, providing direct evidence for β-AR-specific UPR sensor regulation [98]. Future studies should further validate such receptor-UPR sensor specificity across other disease contexts (e.g., metabolic diseases) and explore whether α-adrenergic receptor subtypes exhibit selective regulation of the ATF6 pathway, to refine targeted therapeutic strategies.

### 4.7. Recurrent Hypoglycemia: UPR-Mediated Inhibition of Neurosecretion in the Adrenal Medulla

Research on Sprague Dawley (SD) rats shows that, compared to acute hypoglycemia, recurrent hypoglycemia specifically activates the UPR pathway in the adrenal medulla. Concurrently, recurrent hypoglycemia also induces other pathway abnormalities, including enhanced neuropeptide signaling, imbalances in ion homeostasis, and downregulation of genes related to calcium-dependent vesicular exocytosis. Collectively, these alterations inhibit the neurosecretory function of the adrenal medulla, leading to impaired epinephrine secretion [99]. Thus, UPR activation in the adrenal medulla is a key mechanism by which recurrent hypoglycemia mediates hypoglycemia-associated autonomic failure (HAAF), also providing a potential target for clinical intervention.

Notably, autophagy and the UPR exhibit organ- and cell-type-specific outcomes in chronic SNS activation-related diseases, shaped by tissue/cell-specific functions. In cardiomyocytes, autophagy regulates mitochondrial quality and contractile protein turnover—excessive activation via β-AR/Nox2 signaling leads to cardiac atrophy—while UPR via PERK-ATF4 drives pathological autophagy or apoptosis [51,87,93]. In hepatocytes, autophagy focuses on lipophagy and ER homeostasis with maladaptive accumulation exacerbating steatosis, and UPR’s ATF4-CEBPG-RETREG1 axis governs reticulophagy [74,75,77]. In neurons/microglia, autophagy maintains synaptic function and neuroinflammation balance with flux impairment driving sympathetic overexcitation, and UPR activation in microglia promotes M1 polarization via HMGB1/RAGE [80,85,86]. These differences highlight the cell- and organ-specificity of UPR–autophagy crosstalk in disease progression.

## 5. Targeted Intervention Strategies for Sympathetic Imbalance-Related Diseases: From Molecular Regulation to Systemic Intervention

**Figure 3:**
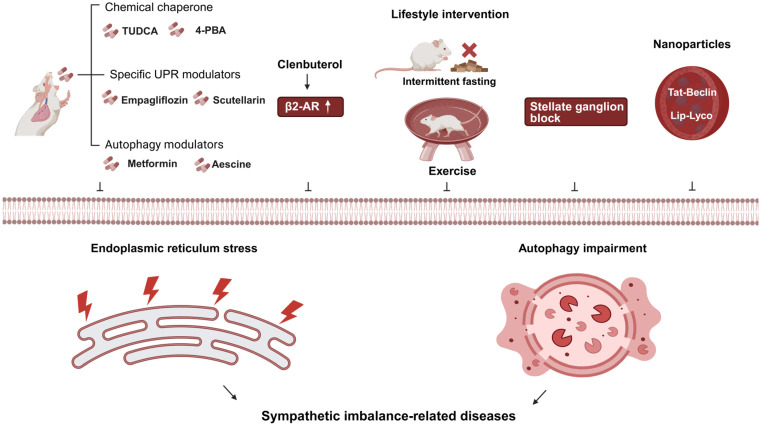
Targeted intervention strategies for sympathetic imbalance-related diseases. Note: In this figure, “↑” denotes increased expression/activity; “⊥” indicates inhibition of the corresponding process (endoplasmic reticulum stress or autophagy impairment); solid black arrows represent the directional regulatory relationship (i.e., the upstream factor leads to/induces the downstream effect). Abbreviations: 4-PBA (4-phenyl butyric acid); β2-AR (beta-2 adrenergic receptor); UPR (unfolded protein response); TUDCA (taurine-conjugated ursodeoxycholic acid); Lip-Lyco (nano-liposomal formulated lycopene); Tat-Beclin (Tat-Beclin1 peptide). Created in BioRender. Bo Xu, Renjun Wang. (2025) https://BioRender.com/mvdqtdb (accessed on 23 January 2026).

This chapter focuses on novel therapeutic strategies for diseases associated with sympathetic imbalance. Centering on the UPR/autophagy pathway as a core molecular hub, it systematically elaborates on multi-level therapeutic approaches. These range from directly modulating cellular stress pathways to indirectly intervening in neural activity to influence downstream UPR/autophagy. The discussion encompasses several key directions: pharmacological strategies that directly target cellular stress pathways [100], neural intervention methods that modulate sympathetic nervous system activity, and the foundational role of lifestyle interventions in restoring neuro-cellular homeostasis. Finally, the chapter explores the emerging potential of nanomedicine in enabling precise and synergistic therapy, as illustrated in Figure 3.

### 5.1. Alleviating Endoplasmic Reticulum Stress: Chemical Chaperones

Studies have confirmed that the orally active chemical chaperones 4-PBA and tauroursodeoxycholic acid (TUDCA) exhibit significant ERS inhibitory effects. In both cellular and animal models, these agents directly reduce excessive ERS activation by enhancing ER adaptive capacity and reducing free cholesterol levels in adipose tissue. Intervention studies in obese diabetic mice demonstrate that these molecules not only normalize hyperglycemia and restore systemic insulin sensitivity but also ameliorate fatty liver disease and enhance insulin action in the liver, muscle, and adipose tissue [101,102]. Furthermore, chemical chaperones can correct ERS-induced adipokine secretion dysregulation, thereby breaking the vicious cycle between metabolic dysfunction and inflammation. This mechanism clarifies the central role of chemical chaperones in intervening in sympathetic imbalance-related metabolic diseases by targeted ERS alleviation, providing a novel drug target and practical rationale for treating type 2 diabetes and obesity-associated metabolic disorders [103].

### 5.2. Specific UPR Pathway Modulators

For myocardial I/R injury, empagliflozin inhibits the PERK/ATF4/Beclin1 pathway, blocks ERS-induced abnormal autophagy, reduces cardiomyocyte apoptosis, and improves cardiac function [9]. In NAFLD, scutellarin suppresses IRE1α/XBP1 pathway hyperactivation. This action upregulates FoxO1-mediated autophagy while reducing lipid accumulation in hepatocytes. XBP1s overexpression attenuates its protective effect, and autophagy inhibitors can negate its benefits, highlighting the regulatory value of UPR–autophagy crosstalk in this context [104]. This category of modulators achieves coordinated regulation of ERS and autophagy by precisely intervening in specific UPR branch signals. It thereby provides more targeted therapeutic directions for diseases such as myocardial injury and NAFLD.

### 5.3. Autophagy Modulators: Promise and Challenges

In the intervention of NAFLD/NASH, several drugs have demonstrated clear autophagy-modulating effects. Sodium-glucose cotransporter 2 (SGLT2) inhibitors, such as empagliflozin, enhance autophagy in hepatic macrophages via the AMPK/mTOR pathway or activate autophagy through the AMPK–TFEB pathway, thereby improving hepatic lipid accumulation and fibrosis [105,106]. The anti-hepatic steatotic effect of metformin depends on TFEB-mediated autophagy activation. It can reverse high-fat diet-induced reductions in TFEB activity and insulin resistance [107]. Additionally, escin activates antioxidative and autophagic responses. Physalin B (PB) activates autophagy and antioxidative signaling; and scoparone enhances autophagic flux in macrophages. All these agents effectively ameliorate NASH-related liver injury and inflammation [108,109,110]. However, the application of autophagy modulators faces challenges. The regulatory mechanisms of autophagy vary among different cell types, necessitating the development of cell-specific targeting strategies [111].

### 5.4. Sympathetic Blockade: Targeting Peripheral Sympathetic Activity to Regulate ERS/Autophagy in Vascular Pathologies

Sympathetic blockade represented by SGB emerges as a promising therapeutic target for vascular-related diseases, complementing existing intervention strategies. As highlighted in Section 4.4, SGB alleviates posthemorrhagic shock-induced vascular hyporeactivity by inhibiting excessive autophagy in vascular smooth muscle cells (VSMCs), reversing PHSML-mediated phenotypic transformation. Moreover, in vascular calcification (VC), SGB suppresses sympathetic overactivation, reduces plasma norepinephrine levels, and attenuates ERS in calcified aortas, thereby ameliorating VSMC osteoblast-like transformation and calcium deposition [112,113]. The beneficial effects of SGB on VC are abolished by the ERS inducer tunicamycin but mimicked by the ERS inhibitor 4-PBA, confirming ERS as a key downstream mediator. These findings validate that targeting peripheral sympathetic activity via SGB regulates ERS/autophagy balance in VSMCs, providing a novel therapeutic direction for vascular pathologies such as VC and post-shock vascular dysfunction.

### 5.5. Indirect Regulation: Potential Benefits of SNS Activity Intervention on Downstream Autophagy

In the regulation of hepatic metabolism, the SNS plays a crucial role in modulating liver autophagy via the β2-AR. β2-AR agonists, such as clenbuterol and epinephrine, can significantly activate autophagy and autophagic flux in hepatocytes, hepatocellular carcinoma cells, and in vivo models. In the field of neuroprotection, NE—the core neurotransmitter of the SNS—activates β2-AR to counteract methamphetamine-induced autophagy dysfunction. Methamphetamine intoxication causes autophagic flux blockage and abnormal translocation of LC3 from autophagic vesicles to the cytoplasm. However, when NE acts directly on methamphetamine-treated PC12 cells, it restores autophagic flux homeostasis and maintains LC3 localization to autophagic vesicles through β2-AR activation. This action effectively antagonizes methamphetamine-induced neuronal injury [22,114]. This mechanism provides a therapeutic direction for degenerative diseases characterized by NE deficiency accompanied by autophagy impairment, suggesting that modulating SNS activity could restore autophagic function.

### 5.6. Lifestyle Interventions: Caloric Restriction and Exercise

Intermittent fasting regimens, such as alternate day fasting and the 5:2 diet, can activate autophagy. In obesity models, caloric restriction combined with exercise reduces body fat percentage and regulates skeletal muscle autophagy [115]. Short-term alternate-day fasting can activate autophagy by upregulating hepatic LC3 and downregulating p62, even without significant weight loss. This activation inhibits lipid synthesis and the expression of inflammatory and apoptotic genes, thereby ameliorating NASH-related liver injury [116]. However, the clinical efficacy of intermittent fasting for MASLD remains heterogeneous, necessitating further clinical studies to clarify its applicable scenarios [117,118].

The regulation of autophagy and UPR by exercise is type and dose dependent. Long-term moderate aerobic or resistance exercise upregulates autophagy-related proteins in aged skeletal muscle, inhibits mTOR, and delays muscle aging. Exercise intervention alone can enhance hepatic autophagy via the AMPK/mTORC1 pathway, impeding the progression from NAFL to NASH and liver fibrosis [119]. In contrast, short-term low-intensity exercise may be insufficient due to failure to reach the activation threshold, whereas high-intensity exercise risks inducing excessive autophagy and causing damage. For middle-aged and elderly populations, long-term combined training can optimize the regulation of skeletal muscle autophagy and UPR pathways, thereby improving exercise performance [120,121].

In summary, both caloric restriction and exercise improve metabolism and tissue aging by physiologically modulating the autophagy–UPR balance. They serve as foundational intervention options for sympathetic imbalance-related diseases. Their core advantages lie in safety and broad applicability. However, their protocols require precise optimization based on disease type and individual tolerance, with clear identification of suitable populations to achieve personalized intervention.

### 5.7. Nanomedicine Strategies: Targeted Delivery and Synergistic Therapy

To address autophagy dysfunction in NAFLD, acid-activated acid-degradable nanoparticles (acNPs) degrade specifically within the acidic environment of hepatocyte lysosomes. This action reconstructs lysosomal acidity and function, restores autophagic flux and mitochondrial homeostasis, and ultimately reverses hyperglycemia and hepatic steatosis [122]. Poly(lactic acid) nanoparticles loaded with Tat-Beclin1 peptide (NP T-B) can specifically accumulate in the liver, enabling potent and sustained autophagy induction at low doses and reducing intracellular lipid accumulation [123].

Nanocarriers also address the poor water solubility of traditional drugs, enabling multi-pathway synergistic intervention. Poly(lactic-co-glycolic acid) (PLGA) nanoparticles loaded with nifedipine (NFD-NPs) achieve sustained drug release in the liver, enhancing autophagic clearance and inhibiting ERS [124]. Orally administered lycopene encapsulated in nanoliposomes (Lip-Lyco) exhibits multiple beneficial effects, including autophagy induction, antioxidant, and anti-inflammatory activities, effectively reversing hepatic steatosis and fibrosis [125]. In summary, nanomedicine, through targeted design and functional integration, efficiently regulates the UPR–autophagy axis and related pathological pathways. It overcomes the delivery limitations of conventional drugs, offering a high-potential, precision therapeutic strategy for metabolic diseases. However, its long-term safety and efficacy require further validation in clinical studies.

## 6. Conclusions and Perspectives

This review demonstrates that an imbalance in the SNS constitutes a common cellular axis for sympathetic imbalance-related diseases by remodeling the crosstalk network between the UPR and autophagy. Chronic SNS overactivation induces metabolic overload and persistent ERS in target organs. This shifts the initially protective UPR–autophagy network toward destructive programs that promote apoptosis, inflammation, and fibrosis. Concurrently, ERS or autophagy dysfunction within central nuclei can directly disrupt autonomic homeostasis, forming a vicious cycle. The elucidation of this mechanism provides a novel theoretical foundation for moving beyond traditional organ-specific therapies and developing synergistic strategies that target this neuro-cellular stress network. These strategies include sympathetic modulation, UPR/autophagy modulators, and nanodelivery systems. However, current mechanistic insights and therapeutic explorations are largely based on animal models such as mice. Human cohort studies and clinical trial evidence necessary for translation are lacking, representing a major bottleneck limiting the conversion of this theoretical framework into clinical applications.

Future research should focus on regulatory specificity and organ interactions. Key priorities include deciphering the direct causal mechanisms underlying SNS-UPR/autophagy crosstalk—such as validating whether catecholamines directly modulate UPR sensors (PERK/IRE1α/ATF6) or autophagy regulators (TFEB/ULK1) via adrenergic receptor-dependent or -independent pathways—and identifying key nodes such as adrenergic receptor subtypes and UPR branches. Notably, the potential sex differences in SNS regulation and ER stress responses remain an understudied area. Current evidence primarily derives from mixed-gender or gender-unspecified studies, and the extent to which biological sex modulates the SNS-UPR/autophagy axis in disease contexts has not been systematically explored. This represents a critical research gap that warrants systematic investigation to guide the development of personalized therapeutic strategies. It is also crucial to define spatiotemporal windows for intervention—for example, targeting ERS in the SFO–PVN circuit during early NAFLD versus focusing on hepatic fibrosis in later stages. Furthermore, elucidating the bidirectional feedback mechanisms between central and peripheral systems is essential to disrupt inter-organ vicious cycles. Building on this foundation, subsequent efforts should explore multi-target combination therapies, optimize nanodelivery platforms, advance clinical translation, and ultimately establish a biomarker-guided precision prevention and treatment system. This integrated approach, leveraging the UPR–autophagy network, holds promise for driving breakthroughs in chronic disease research.

## Figures and Tables

**Table 1:** Organ-specific mechanisms of UPR–autophagy crosstalk.

Disease Model	Core Regulatory Axis	Core Mechanism	Functional	References
Diabetic nephropathy mice model	ATF4–HO-1–Autophagy PERK–ATF4 HO-1	Transcriptional induction of HO-1 expression by ATF4	Enhances autophagic flux initiates protective autophagy, clears damage, and inhibits podocyte apoptosis	[73]
MASLD mouse model	ATF4/CEBPG-RETREG1	Acute phase: ATF4/CEBPG ↑ → RETREG1 ↑ Chronic Phase: ATF4/CEBPG ↓ → RETREG1 ↓	Acute: Initiates reticulophagy, maintaining homeostasis Chronic: autophagy defect, persistent ERS, aggravated injury	[74]
Liver disease HepaRG cell line	PERK–ATF4–CHOP	Excessive activation of the PERK–ATF4 pathway drives CHOP expression	Induces pathological overactivation of autophagy and hepatocyte injury	[75]
Mouse model of acute myocardial infarction	VEGF-A–ROS–ERS–Autophagy	VEGF-A activates ERS and autophagy via ROS	Promotes angiogenesis and mediates repair in the acute ischemic phase	[71]
β_1_-AAs active immunization rat model	β_1_-AR Autoantibody–ERS/Autophagic flux	Antibodies continuously activate ERS but inhibit autophagic flux	ERS decouples from autophagy, disrupting cardiomyocyte homeostasis	[72]
Myocardial infarction animal model	Thbs1–PERK–ATF4 ATP–P2X7–Nox4–PERK–ATF4	Thbs1 or ATP activates the PERK–ATF4 pathway via P2X7–Nox4	Drives excessive autophagy, leading to myocardial atrophy or exacerbating reperfusion injury	[51,76]
Diabetic pancreatic islet model	CD47-TSP1-UPR -Autophagy	Metabolic stress upregulates CD47-TSP1; inhibiting CD47 signaling improves UPR function and restores autophagic homeostasis	Maintains β-cell homeostasis, reduces senescence/apoptosis, and preserves insulin secretion	

Abbreviations: In the table, “↑” indicates an increase in expression/activity, and “↓” indicates a decrease in expression/activity. UPR (unfolded protein response), ATF4 (activating transcription factor 4), HO-1 (heme oxygenase-1), MASLD (metabolic dysfunction-associated steatotic liver disease), CEBPG (CCAAT/enhancer-binding protein gamma), RETREG1 (reticulophagy regulator 1), ERS (endoplasmic reticulum stress), CHOP (C/EBP homologous protein), VEGF-A (vascular endothelial growth factor-A), ROS (reactive oxygen species), β_1_-AR (β_1_-adrenergic receptor), Thbs1 (thrombospondin 1), P2X7 (purinergic receptor P2X7), Nox4 (NADPH oxidase 4), PERK (protein kinase R-like endoplasmic reticulum kinase).

**Table 2:** Regulatory roles of sympathetic/autophagy-related factors in organ-specific pathophysiology and mechanisms.

Disease Model	Influencing Factors	Target Organ	Pathophysiological Indicators	Molecular Mechanisms	References
heart failure mouse model	NADPH oxidase 2	Myocardium	Sympathetic Nerve Activity (SNA) ↑; myocardial NE nerve fibers ↓ Cardiac atrophy; cardiac function decline	ROS ↑; PGP9.5, GAP43 ↓ LC3-II, Beclin1 ↑	[87]
Rat IBAT temperature stimulation model	SNS	BAT	BAT atrophy (25 °C) BAT hypertrophy (4 °C)	Forkhead box O, phosphorylation ↑	[88]
Injury model of PC12 cells	NE	PC12 cells	Autophagy flux block LC3 mislocalization	β2-adrenergic receptors ↓	[22]
Rat hemorrhagic shock model	Stellate Ganglion Block (SGB)	Spleen	CD4^+^ T cell proliferation ↓ Immune function inhibition Autophagy ↑	PHSML; LC3-II/I; Beclin1 ↑ PI3K/Akt pathway ↓	[89]
Conscious rat hemorrhagic shock model	SGB	Vascular smooth muscle cells	Vascular hyporeactivity Phenotypic transformation (contractile → synthetic)	PHSML ↑ LC3-II/I; Beclin1; MMP-2 ↑	[23]
Conscious rat hemorrhagic shock model	SGB	Intestinal mucosa	Intestinal barrier damage ERS; Autophagy ↑	ATF6α, PERK, IRE1α, LC3-II, Beclin1 ↑ Zonula occludens-1, occludin ↓	[90]
Clinical model of type 2 diabetes mellitus patients	SNS	Monocytes in the cardiovascular system	NE; C-reactive protein ↑ Atherosclerotic progression markers ↑	GRP78/BiP; EIF2α; XBP1 ↑ TLR2/4; NFκB; Beclin1 ↑	[91]
Rat model of myocardial ischemia/ reperfusion	SNA	Cardiomyocyte	Ventricular arrhythmia score ↑ HRV: LF/HF ratio ↑ left ventricular ejection fraction ↓ Myocardial infarct size ↑	LC3B ↓, P62 ↑ Cleaved caspase-3 ↑ p-Cx43 ↓, Np-Cx43 ↑	[92]

Abbreviations: In the table, “↑” indicates an increase in expression/activity, and “↓” indicates a decrease in expression/activity. ATF6α (activating transcription factor 6α); GRP78/BiP (glucose-regulated protein 78/binding immunoglobulin protein); HRV (heart rate variability); IRE1α (inositol-requiring enzyme 1α); LC3 (microtubule-associated protein 1 light chain 3); LF/HF ratio (Low-Frequency/High-Frequency ratio); MMP-2 (matrix metalloproteinase 2); NE (norepinephrine); NFκB (nuclear factor kappa-B); PERK (protein kinase R-like endoplasmic reticulum kinase); PGP9.5 (protein gene product 9.5); PI3K/Akt (phosphatidylinositol 3-kinase/protein kinase B); PHSML (post-hemorrhagic shock mesenteric lymph); ROS (reactive oxygen species); TLR2/4 (toll-like receptor 2/4); XBP1 (X-box binding protein 1).

## Data Availability

The original contributions presented in this study are included in the article. Further inquiries can be directed to the corresponding authors.
